# A Compend of Human Physiology

**Published:** 1890-07

**Authors:** 


**A Compend of Human Physiology.** Especially adapted for the use of Medical Students. By ALBERT P. BRUBAKER, A. M., M.D., Demonstrator of Physiology in the Jefferson Medical College; Professor of Physiology, Pennsylvania College of Dental Surgery, etc. Fifth edition, revised and enlarged, with new illustrations and a Table of Physiological Constants. Philadelphia: P. Blakiston, Son & Co. 1889.

This is one of the Blakiston Series of quiz-compends, and is no longer strange to the profession. When a book reaches the necessity of a fifth edition its claim to be called standard is tolerably well established. This work is especially designed for students, and many teachers recommend it to their pupils. It admirably fills the place which it is put forward to occupy.

